# Fetal Cyclophosphamide Exposure Induces Testicular Cancer and Reduced Spermatogenesis and Ovarian Follicle Numbers in Mice

**DOI:** 10.1371/journal.pone.0093311

**Published:** 2014-04-01

**Authors:** Paul B. Comish, Ana Luiza Drumond, Hazel L. Kinnell, Richard A. Anderson, Angabin Matin, Marvin L. Meistrich, Gunapala Shetty

**Affiliations:** 1 Department of Experimental Radiation Oncology, The University of Texas MD Anderson Cancer Center, Houston, Texas, United States of America; 2 Department of Genetics, The University of Texas MD Anderson Cancer Center, Houston, Texas, United States of America; 3 MRC Centre for Reproductive Health, Queens Medical Research Institute, University of Edinburgh, Edinburgh, United Kingdom; University Hospital of Münster, Germany

## Abstract

Exposure to radiation during fetal development induces testicular germ cell tumors (TGCT) and reduces spermatogenesis in mice. However, whether DNA damaging chemotherapeutic agents elicit these effects in mice remains unclear. Among such agents, cyclophosphamide (CP) is currently used to treat breast cancer in pregnant women, and the effects of fetal exposure to this drug manifested in the offspring must be better understood to offer such patients suitable counseling. The present study was designed to determine whether fetal exposure to CP induces testicular cancer and/or gonadal toxicity in 129 and in 129.MOLF congenic (L1) mice. Exposure to CP on embryonic days 10.5 and 11.5 dramatically increased TGCT incidence to 28% in offspring of 129 mice (control value, 2%) and to 80% in the male offspring of L1 (control value 33%). These increases are similar to those observed in both lines of mice by radiation. *In utero* exposure to CP also significantly reduced testis weights at 4 weeks of age to ∼70% of control and induced atrophic seminiferous tubules in ∼30% of the testes. When the *in utero* CP-exposed 129 mice reached adulthood, there were significant reductions in testicular and epididymal sperm counts to 62% and 70%, respectively, of controls. In female offspring, CP caused the loss of 77% of primordial follicles and increased follicle growth activation. The results indicate that i) DNA damage is a common mechanism leading to induction of testicular cancer, ii) increased induction of testis cancer by external agents is proportional to the spontaneous incidence due to inherent genetic susceptibility, and iii) children exposed to radiation or DNA damaging chemotherapeutic agents *in utero* may have increased risks of developing testis cancer and having reduced spermatogenic potential or diminished reproductive lifespan.

## Introduction

In the past 50 years, the incidence of testicular germ cell tumors (TGCTs) among Caucasian men age 15–40 years, the group in which these malignancies occur most frequently, has increased 3-fold [1]. Men's sperm production has also declined constantly over the last six decades [2]. Both of these adverse outcomes are suggested to be the result of prenatal exposure to environmental agents, mainly endocrine disruptors [3]. Nevertheless, there are no unequivocal studies showing endocrine disruptors to be the causative factors behind an increase in TGCT incidence [4], [5]. Furthermore, there is a rise in early menopause (premature ovarian failure) in women that seems to be largely attributable to increased survival of cancer patients treated with radiotherapy and chemotherapy as children or young women [6]; however, the contribution of fetal exposures have not been examined.

The 129 mouse model [7], which has a 5% incidence of testicular teratomas, is the only animal model of human TGCTs, and although whether results using this model can be extrapolated to tumors arising in young men is still a matter of some debate, there are many similarities. These teratomas are histologically and developmentally similar to human teratomas. In both humans and mice, teratomas arise during early postnatal development due to a failure of the pluripotent PGCs, arriving at the genital ridge, to differentiate into gonocytes committed to spermatogenesis [7], [8]. Further, both are predisposed by mutations in the Kitl or Dmrt1 genes [9]–[12], and both appear to involve epigenetic changes in their etiology [13], [14].

In a previous study, we found that the TGCT incidence of mice exposed to low doses of ionizing radiation *in utero* was dramatically increased above than that of control mice [5]. We also found that, non–tumor-bearing testes were significantly smaller at 28 days of age, compared with unirradiated counterparts, indicating reduced spermatogenesis. Extrapolation of these findings to human conditions led us to hypothesize that radiation exposure *in utero* may increase males' risks of TGCT and infertility. However, given the relatively low radiation doses that women of childbearing age currently receive, it is unlikely that radiation *in utero* has been responsible for the global elevation in the TGCT incidence and decline in sperm counts. Rather, exposure to a chemical whose primary biological mechanism of action is similar to that of radiation could be responsible for these adverse outcomes.

Because radiation acts by DNA damage, we hypothesized that DNA-damaging chemicals, particularly the highly carcinogenic alkylating agents, may be candidates for the induction of TGCT. We chose to examine the effects of cyclophosphamide (CP) because it has been widely studied and is used to treat pregnant women such as those with breast cancer as part of the FAC (5-fluorouracil, Adriamycin, cyclophosphamide) combination chemotherapy regimen [15]. CP could be carcinogenic to the embryo as it has high transplacental transfer in non-human primates [16], induces DNA strand breaks in mouse embryos [17], and causes secondary malignancies in cancer patients [18]. In addition CP is a major reproductive toxicant in male and female mice [19], [20] and prepubertal and adult humans exposed to CP can develop permanent azoospermia and premature ovarian failure [21], [22]. However, its effects on carcinogenesis and reproductive function after fetal exposures have not been yet investigated.

We therefore hypothesized that *in utero* CP exposure may be carcinogenic to the testis and induce reproductive toxicity in both sexes. We used the 129 as well as the more sensitive 129.MOLF-L1 testis cancer mouse model for testing induction of TGCT by this chemical and also to test testicular toxicity. Only 129 mice were used to test the ovarian toxicity. Exposures were done at E10.5 and 11.5 which is known to be a sensitive stage for induction of testicular cancers [5], [7]. In addition there are only 1,000 to 3,000 germ cells at these ages and the stromal cells are just being formed, making both the gonadal germ cells and stromal cells targets for reproductive toxicity.

## Materials and Methods

### Mice and breeding

Inbred congenic 129.MOLF-L1 mice, referred to as L1, had been derived by crossing 129S1/SvImJ and MOLF/Ei inbred mice of the *Mus musculus molossinus* subspecies [23]. About 30% of L1 males develop spontaneous testicular tumors. In addition, 129S1/SvImJ (Jackson Laboratories, Bar Harbor, ME) and 129/S5 (previously obtained from Taconic and maintained in our laboratory) mice, both of which have ∼5% incidence of spontaneously developed TGCTs were also used. No major significant differences between the results with the two 129 sublines were observed, so that the data could be combined.

All experimental procedures were approved by the MD Anderson Cancer Center Institutional Animal Care and Use Committee with approved protocol numbers 110712632 and 110712633. The mice used in the study were housed in facilities at MD Anderson that are registered by the U.S. Department of Agriculture and accredited by the American Association for the Accreditation of Laboratory Animal Care.

### Treatments

Timed matings were performed with pairs of L1 or 129 mice.

Pregnant females were irradiated with two doses of 0.8 Gy of ^6^°Co-gamma radiation given on days 10.5 and 11.5 of their pregnancy (E10.5 and E11.5) as described previously [5]. The male offspring of 129 and L1 dams from the same breeding colonies that did not receive any of these treatments or manipulations were analyzed as controls.

Pregnant 129 or L1 mice were injected (ip) with CP, dissolved in saline at doses of 7.5 mg/kg on days 10.5 and 11.5 of pregnancy. The offspring from dams of the same breeding colony that received saline on these days were used as controls.

### Analysis

Male offspring exposed to cyclophosphamide or radiation *in utero* and control male offspring were euthanized at 4 weeks of age, because most testicular tumors are readily observable at this age [23]. To determine the effects of *in utero* cyclophosphamide exposure on spermatogenesis in adults, some offspring of 129Sv mice were kept until 8 weeks of age and then euthanized. The testes were weighed and then fixed in Bouin's solution for histologic examination and tumors were identified by the analysis of hematoxylin and eosin-stained sections, as described previously [5].

In sections from normal non-tumor-bearing testes of 4-week-old mice, the numbers of moderately large atrophic tubules without any germ cells (Sertoli-only tubules), observed mostly in the interior region of a single mid-cross section of the testis, were counted. The small atrophic tubules present in the peripheral region near the rete testes were excluded from this analysis. Occasional testes in the control or treatment groups that had >20% atrophic tubules, possibly due to an inherent abnormality, were excluded from this analysis or from testis weight averages.

Historical data from a previous study on the induction of TGCT in *in utero* irradiated L1 mice [5] were also used for comparison.

Sperm production was assessed in adult 129 mice that did not bear tumors. One of the testes was homogenized after weighing and sonicated. The sperm heads were counted in a hemocytometer [24]. For epididymal sperm counts, both cauda epididymides were minced separately in 1 ml PBS and incubated at 37°C for 30 min, and the suspension was passed through a 80-μm pore size metal filter. Sperm were counted using a hemocytometer.

To analyze the effects of *in utero* CP exposure on ovarian follicular development, female pups were euthanized at 7 days after birth and the ovaries were fixed in Bouin's fluid. The ovaries were paraffin-embedded and serially sectioned at 5-μm thickness and slides were stained with hematoxylin and eosin.

To determine changes in ovarian volume, every 10th slide was used to generate an area measurement using the Image-Pro Plus software. These area measurements were then used to calculate the ovarian volume based on the thickness of the sections and the total number of slides comprising the ovary.

To count different types of follicles, one section from every 10th slide was imaged using a 40× objective and tiled [25]. Follicles were classed as either primordial (having a single flattened layer of granulosa cells); primary transitional (having some, but not all, of the granulosa cells already cuboidal); primary (having a single layer of cuboidal granulosa cells); or secondary (having more than one layer of cuboidal granulosa cells). The total number of follicles/ovary were calculated from the raw follicle counts per section using the Abercrombie equation [26] to correct for follicle size.

### Statistical Analysis

Numerical data are presented as mean ± standard errors of the means. The significance of differences in continuous variables (e.g. litter size, weights, sperm counts, tubular or follicular numbers) between exposed and control mice were evaluated by a Student's *t*-test. The significance of the differences in categorical variables (e.g. production of progeny, presence of tumors or atrophic tubules) between exposed and control mice were determined using the Fisher's exact test. *P* values <0.05 were considered statistically significant. A computer-assisted statistics program (IBM SPSS version 19) was used.

The data for the offspring of the CP controls (*in utero* saline-treated mice) and radiation-controls (offspring of untreated mice) were not significantly different (with one exception indicated in Table S1) and were pooled together.

## Results

Pregnant 129 (129S1/SvImJ and 129S5) and L1 mice were treated with two doses of CP, one at each of days 10.5 and 11.5 of pregnancy. Initial studies tested doses of 25 and 10 mg/kg given on both days, but, although all females survived, 0/15 and 0/7 plug-positive females, respectively, produced offspring. When the CP dose was reduced to 7.5 mg/kg/day, 24% and 31% of the successfully mated 129 and L1 mice, respectively, produced progeny (Table 1). The percentage of progeny-producing CP-treated 129 dams (24%) was significantly lower than that of the control dams (50%) but was not significantly different from that of the radiation-treated dams (33%). However, CP or radiation did not affect litter sizes. In addition, mice exposed to radiation or CP did not show any major teratogenic effects, except that most of the mice exposed to CP *in utero* had kinks in their tails (Fig. S1). Overall, there were small but significant reductions in the body weights of the mice after fetal exposure to either CP and radiation.

**Table 1:** Effect of cyclophosphamide (CP) or radiation on days 10.5 and 11.5 after mating on breeding efficiency of 129 and L1 mice.

Mouse strain	Treatment	Vaginal plug positive mice	Produced progeny a	Average litter size	Body weight of male offspring at 28 days (g) b
129 c	Control	20	10 (50%)	4.9±0.5	14.8±0.3
129 c	CP	130	31 (24%) d	4.5±0.4	13.3±0.4^e^
129 c	Control	ND	ND	5.1±0.4	16.3±0.4
129 c	Radiation	52	17 (33%)	5.4±0.7	14.4±0.3^e^
L1	Control	12	7 (58%)	5.1±0.6	16.8±0.5
L1	CP	39	12 (31%)	5.4±0.4	12.7±0.3^e^
L1	Control f	ND	ND	6.4± 0.7	16.8±0.4
L1	Radiation f	28	15 (54%)	5.3±0.6	14.4±0.5^e^

Abbreviation: ND, no data.

a Values given as absolute number and percentage of total vaginal plug-positive females.

b The number of male offspring per group ranged from 19 to 51.

c Data from 129S5 and 129S1/SvImJ mice were pooled.

d Significantly different between treated and control mice (*P*<0.05; Fisher's exact test).

e Significantly different between treated and control mice (*P*<0.05; *t* test).

f Historical data from previous study [5] were used for comparison.

**Figure 1:**
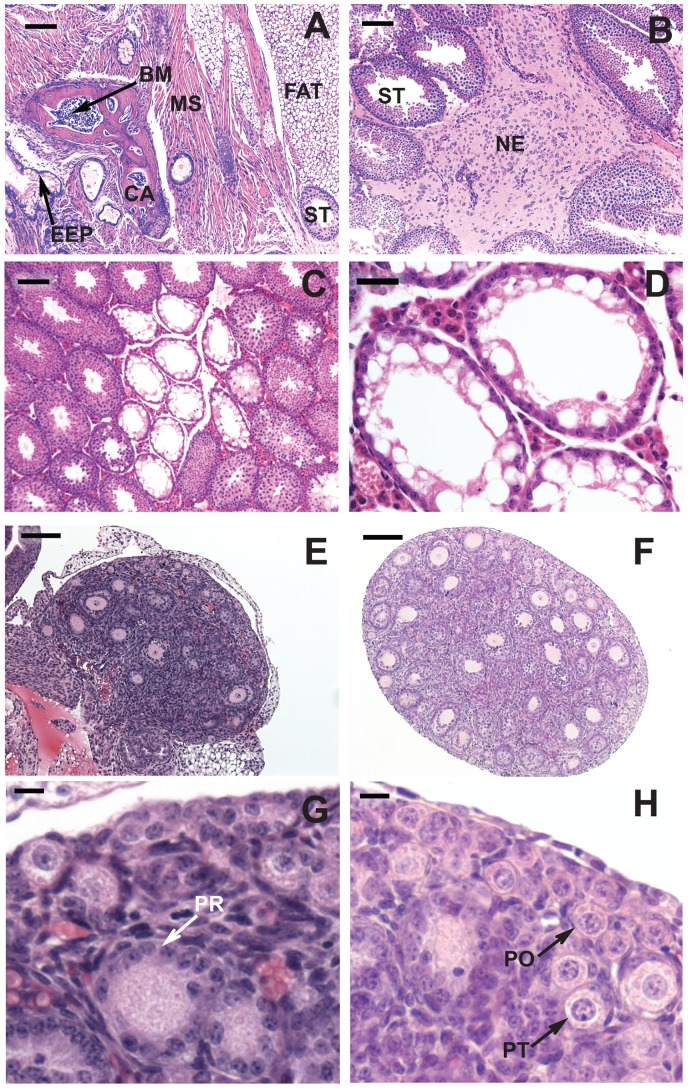
Histology of testes and ovaries from 129 mice exposed to cyclophosphamide at 7.5 mg/kg/day on E10.5 and 11.5. (A-D) Testes from 4-week-old mice. (A) TGCT characterized as a teratoma originating from multiple dermal layers; (B) TGCT containing only neuroepithelial cells. Abbreviations: BM: bone marrow; CA: cartilage; EEP: endodermal epithelium; NE: neuroepithelial cells; MS: muscle; ST: seminiferous tubule. (C) non-TGCT-bearing testis showing active and atrophic tubules. (D) High magnification of atrophic tubules containing only Sertoli cells. (E-H) Ovaries from 7-day-old mice. (E, G) From a mouse treated on E10.5 and 11.5 with 7.5 mg CP/kg/day. (F, H) Control ovary of the same age. G and H are the magnified views from portions of E and F respectively, showing primordial (PO), primary-transitional (PT) and primary (PR) follicles. The bar represents 100 μm in A, B, C, E & F; 30 μm in D; 10 μm in G and H.

Testes were harvested from the male offspring and the presence and types of tumors was determined by histological examination (Fig. 1 A & B). Exposure to CP during pregnancy significantly (*P*<0.001) increased TGCT incidence in the male offspring of 129 mice from a control value of 2% to 28% and in L1 mice from 33% to 80% (Table 2). In both mouse strains, the higher incidence of tumors among mice exposed to CP *in utero* was similar to that of mice exposed to radiation *in utero*. The numbers of mice with unilateral or bilateral tumors followed a binomial distribution, indicating that the occurrence of a tumor in each testis was an independent event. Thus expressing the data on a per testis basis, CP induced TGCTs in 18% of the testes in 129 mice and in 64% of the testes in L1 mice. Most of these tumors were teratomas (Fig. 1A); the rest contained only neuroepithelial tumor cells (Fig. 1B). Compared with those of control mice, the TGCT-bearing testes of L1 mice, but not 129 mice, exposed to CP or radiation were significantly heavier. This suggests that both CP- and radiation induced multiple tumor foci in some of the testes of L1 mice. To determine whether minor differences in genetic makeup affected tumor induction, we compared these results on induced TGCT incidence of 129S1/SvImJ mice to those of 129S5 mice (Table S1). There was only a marginally significant difference (*P* = 0.04) with higher incidence of TGCTs in CP-treated 129S5 mice than in CP-treated 129S1/SvImJ mice.

**Table 2:** Increased testicular germ cell tumor incidence (TGCT) in 129 and L1 mice exposed *in utero* to cyclophosphamide (CP) or radiation.

Mouse strain	Treatment	No. males analyzed	Overall TGCT per mouse a	Bilateral TGCT per mouse a	No. testes analyzed	TGCT per testis a	Identified as teratomas a	Weight of testis with TGCT (mg)
129 b	Control	82	2 (2%)	0 (0%)	164	2 (1%)	2 (100%)	75±10
129 b	CP	53	15 (28%) c	4 (8%)^c^	106	19 (18%) c	13 (68%)	88±12
129 b	Radiation	34	11 (32%) c	0 (0%)	68	11 (16%) c	9 (82%)	74±16
L1	Control	46	15 (33%)	2 (4%)	92	18 (20%)	13 (72%)	80±9
L1	CP	20	16 (80%) c ^,^ d	9 (47%) c	39 e	25 (64%) c	21 (84%)	130±24 f
L1	Radiation g	32	32 (100%) c ^,^ d	19 (59%) c	64	51 (80%) c	48 (94%)^c^	137±21 f

a Values given as absolute number and percentage of mice, testes, or tumors analyzed.

b Data from 129S5 and 129S1/SvImJ mice were pooled.

c Significantly different between treated and control mice (*P*<0.05; Fisher's exact test).

d Significantly different between CP-exposed and irradiated mice of the same strain (*P*<0.05; Fisher's exact test).

e The odd number is due to the presence of only one testis in one of the mice analyzed, which was not considered for bilateral TGCT analysis.

f Significantly different between treated and control mice (*P*<0.05; *t* test).

g Data from a previous study [5] were included for comparison.

Furthermore, at 28 days of age the weights of tumor-free testes of 129 and L1 mice exposed to CP *in utero* were reduced to 74% and 72%, respectively, of those of the control mice (Table 3); similar to the decrease observed with 2×0.8 Gy radiation *in utero*. This reduction in testis weight did not appear to be the result of body weight loss, as the ratio of the testis weight to body weight of the toxicant-exposed mice was also significantly lower than that of the control mice. Atrophic Sertoli cell-only tubules were frequently observed in the groups of treated mice (Fig. 1 C & D) but rarely observed in the controls. For example, about 30% of the testes from the CP- or radiation-treated 129 mice had atrophic tubules with an average of 6 per testis section (Table 3). In contrast atrophic tubules were observed in only 4% of the testes from control mice, with an average of 1.6 atrophic tubules per section. Even when *in utero* CP-exposed 129 mice reached adulthood, the weights of the testes remained reduced to 78% of control values (Table 4). Reductions in the counts of testicular and epididymal sperm to 62% and 70%, respectively, of controls were observed in these mice after *in utero* CP exposure, demonstrating that germ cell loss was primarily responsible for the reduction in testis weights.

**Table 3:** Reproductive toxicity in male 129 and L1 mice exposed to cyclophosphamide (CP) or radiation *in utero*.

Mouse strain	Treatment	No. analyzed testes without TGCT	Weight of testis at 28 days (mg)	Testes with atrophic tubules a	No. atrophic tubules per cross section
129 b	Control	159	57±1	6 (4%)	1.7±0.2
129 b	CP	87	42±1 c	25 (32%) d	5.6±0.8 c
129 b	Radiation	57	38±1 c	18 (32%) d	4.8±0.8 c
L1	Control	72	60±1	4 (6%)	1.5±0.5
L1	CP	13	43±2 c	5 (38%) d	2.4±0.5
L1	Radiation e	13	37±1 c	2 (15%)	3.5±0.5

a Values given as absolute number and percentage of testes analyzed

b Data from 129S5 and 129S1/SvImJ mice were pooled.

c Significantly different between treated and control mice (*P*<0.05; *t-*test).

d Significantly different between treated and control mice (*P*<0.05; Fisher's exact test).

e Data from previous study [5] was included for comparison.

**Table 4:** Reduction of testis weights and sperm counts in 8-week-old 129S1/SvImJ mice exposed to cyclophosphamide (CP) *in utero*.

Treatment	Testis weight (mg)	Sperm head count/testis	Sperm count/cauda epididymis
Control (n = 5)	97±2	29 (±2) × 10^6^	10.2 (±0.8) × 10^6^
CP (n = 4)	75±1 a	18 (±2) × 10^6^ a	7.1 (±0.5) × 10^6^ a

a Significantly different between treated and control mice (*P*<0.01; *t*-test).

In female offspring, *in utero* exposure to CP significantly reduced the volume of ovaries to a value of 5.5±0.6×10^7^ μm^3^, compared to 13.0±1.4×10^7^ μm^3^ in controls (*P* = 0.001; Fig. 1 E & F). In these mice, the numbers of primordial follicles per ovary was only 23% of control mice (Table 5 and Fig. 1 G & H). Interestingly, although mice exposed to CP *in utero* had lower numbers of growing (transitional primary, primary, and secondary) follicles than controls, the ratio of the total number of developing follicles to the number of primordial follicles was increased from 0.23 in controls to 0.62 in *in utero* CP-exposed offspring (*P*<0.05).

**Table 5:** Reduction of the number of primordial and developing follicles in the ovaries of 7-day- old 129S5 mice exposed to cyclophosphamide (CP) *in utero*.

Follicle type	Control (number per ovary) (n = 4)	CP (number per ovary) (n = 5)	CP ÷ control
Primordial	10,960±1,056	2,532±505 a	23%
Primary-transitional	1,176±179	698±97 a	59%
Primary	380±99	238±48	63%
Secondary	863±96	420±66 a	49%
Total developing ÷ primordial	0.23±0.03	0.62±0.12 a	

a Significantly different between treated and control mice (*P*<0.05; *t*-test).

## Discussion

In the present study we demonstrated for the first time that chemical exposure during fetal development can induce TGCTs. We also demonstrated that *in utero* exposure to radiation or CP dramatically increases the incidence of testicular tumors in 129 mice, thereby extending our previous findings using L1 mice, which are highly susceptible to testicular tumors [5], to a commonly used strain of mice that is only moderately susceptible to such tumors. The qualitative and quantitative similarities in the induction of TGCTs between the mice exposed to radiation *in utero* and those in mice exposed to CP *in utero* indicate that radiation and CP have similar mechanisms of testicular tumorigenesis. We also showed that these *in utero*-exposures resulted in marked reductions in germ cell numbers in ovaries of female offspring and in the testes of male offspring that did not develop tumors.

The time points for exposure to potential carcinogenic agents for induction of TGCTs were initially chosen at E10.5 and E11.5 [5] since those are the earliest days just after the PGCs colonize the fetal gonad and are undergoing extensive epigenetic changes [14]. Furthermore, the PGCs still express pluripotency markers and have the ability to form teratomas in transplantation assays [7], which are lost by day E13.5. The demonstration of susceptibility to induction of TGCTs during this time window for two agents, CP and radiation, and for two strains of mice, 129 and L1, supports this choice. Although other ages have not yet been investigated, it is most likely that the window of sensitivity to induction of TGCTs by radiation and CP is limited to E10.5 to E12.5 in mice, which is the period in mice during which the PGCs are in the testes and are pluripotent, and not yet committed to germ cell lineages.

Previous studies have shown that single exposures of low-dose (∼1.5 Gy) radiation delivered between E14.5 and birth cause long-term damage to spermatogenesis in both mice [27] and rats [28]. The findings of the present study suggest that this window of sensitivity to radiation is even longer, extending to as early as E11.5 or E10.5. The effects we observed demonstrate that the prenatal testes are more sensitive to long-term effects of radiation exposure than adult testes are.

Although CP is a known teratogen and reproductive toxicant to mammals after postnatal exposure, its prenatal effects on spermatogenesis are relatively unknown. One study [29] indicated that CP given to rats at day 12 of pregnancy affected the migration of PGCs in the fetuses but did not follow the postnatal development of the gonads. Our finding that a total dose of CP of 15 mg/kg has long-term effects on spermatogenesis indicates that male gonads at E10.5–E11.5 are much more sensitive to CP than adult testes are [30]. Because spermatogenesis was sensitive to radiation from E10.5 to birth, it is likely that spermatogenesis is also sensitive to CP at these times. However, this remains to be tested.

The sensitivity of oocyte and follicular numbers to CP treatment was expected since the treatment that affected spermatogenesis was given at E10.5-E11.5 when the PGCs were in undifferentiated gonads. If depletion of PGCs was not compensated through extra proliferation of oogonia, this must result in fewer oocytes in the ovary. Studies investigating the exposure of female rat fetuses to low doses of radiation [31] or busulfan [32] have shown that the ovary is sensitive to these agents between E13.5 and 17.5. Hence, it is likely that the ovary will be sensitive to CP throughout the latter half of fetal development.

Because CP, like radiation, is a DNA-damaging agent, DNA damage may be a common mechanism that leads to TGCT formation and germ cell loss in embryonic gonads. The damage produced by radiation and that caused by CP have some common features. Radiation, either directly or through free radical/reactive oxygen species, indirectly causes mainly single- and double-strand breaks but also creates some base damage [33]. The CP metabolite phosphoramide mustard forms adducts and interstrand cross-links with DNA [34] and the metabolite acrolein produces reactive oxygen species and DNA adducts [35]. The DNA damage likely kills the PGCs leading to deficiencies in spermatogenesis and ovarian reserves. In addition, in order to repair or bypass the DNA damage, a DNA-damage-response pathway is activated and the relationship of genes in this pathway to human testicular cancer has been demonstrated in a genome-wide association study [36]. One possibility for the mechanism by which the DNA-damage response causes TGCT is that it interferes with the extensive epigenetic changes occurring within the PGCs between E10.5 and 11.5 that are involved in the loss of pluripotency and commitment to germ cell differentiation [14].

The role of genetic background in the induction of TGCT by exogenous agents and its relation to spontaneous incidence was assessed by comparing induction in 129 *vs.* L1 mice. Since tumors were present in a high percentage of exposed testes and the increased size indicated multiple tumor foci, it was necessary to estimate the numbers of tumor foci per testis, taking into account the Poisson distribution. The numbers of foci were much greater in treated L1 mice (0.81 and 1.38, respectively, for CP and radiation) than in 129 mice (0.19 and 0.16, respectively) (Table S2). The data suggest that the increase in induced TGCTs by DNA damaging agents is proportional to the spontaneous incidence, and that the susceptibly to induction of TGCT in humans would depend on the spontaneous incidence in different ethnic backgrounds [37].

The mechanism by which radiation and CP cause spermatogenic defects likely involves the killing of the PGCs or supporting Sertoli cells. Whereas smaller testis could result from either fewer Sertoli cells or spermatogonial stem cells to fill the niches, the presence of atrophic tubules with morphologically normal Sertoli cells (Fig. 1C & D) indicates that germ cells loss is the predominant effect.

The reduction in ovarian follicle numbers we observed also could have resulted from CP depleting the PGCs in undifferentiated gonads. In addition, the higher ratio of developing follicles to primordial follicles in CP-exposed mice compared to controls demonstrates an increase in the rate of follicle recruitment, as has been observed in rats in which exposure to busulfan *in utero* depleted the primordial follicle pool [38]. This enhanced activation of follicle recruitment may be a result of lower concentrations of inhibitory factors produced from the lower number of primordial follicles themselves [39] or from lower amounts of anti-Mullerian hormone produced from reduced numbers of developing preantral follicles [40]. The increased rate of follicle recruitment would even further deplete the primordial follicle pool as these mice aged; thus CP treatment *in utero* prior to entry into meiosis may be a potential model for premature ageing of the ovary.

The results of the present study have clinical importance, as many pregnant women are diagnosed with breast cancer or other life-threatening cancers requiring immediate treatment, which usually includes DNA-damaging alkylating agents such as CP. Within the last few years several groups have reported on more than 400 children of women treated with chemotherapy during the second and/or third trimesters [41]–[45]. The birth defect rate and general health and growth in these children are not significantly different from those of children whose mothers were not treated with chemotherapy during these times. These children included those of more than 100 women who received CP doses of about 2,000 mg/m^2^, a part of FAC chemotherapy for breast or gynecological cancer during pregnancy at The University of Texas MD Anderson Cancer Center [15] (J.T. Litton, unpublished communications). However, most of the children from the above studies are currently prepubertal and still have not reached the age at which the overt signs of possible adverse outcomes such as testicular pathogenesis or ovarian insufficiency become apparent.

In humans the window of susceptibility of PGCs to tumorigenic effects of DNA damaging agents is likely more prolonged than in rodents. The reason being, in humans the transformation of potentially pluripotent PGCs to committed germ cells, which starts after the PGCs have arrived at the testes at 6 weeks of fetal development, continues gradually throughout the rest of pregnancy and even after birth [46]. Thus, although chemotherapy is given during the second and third trimesters, the germ cells are still susceptible to the toxic and tumorigenic effects of chemotherapy.

It is important to consider how to extrapolate the possible detrimental effects of CP found in the present study to human. If human males exposed to CP *in utero* have a 6-fold increase in testicular cancer incidence, as was observed in the mice in the present study, the cumulative incidence of testicular cancer in men up to age 44 years would be increased from about 3 per 1000 to about 18 per 1000 males [47]. To detect such an effect, a large study is required with long-term follow up, as most of the testicular cancers occur after puberty. However, because the cumulative CP dose to pregnant women (2,000 mg/m^2^) [15] is 40 times that given to mice in the present study (50 mg/m^2^), the testicular cancer incidence could be higher and thus more readily detectable. In any case, the effects of CP on reproductive function may be more widespread and easier to detect in limited populations.

Because the effects, if any, of *in utero* exposure in humans remain uncertain, physicians should use noninvasive markers of possible negative outcomes. Spermatogenic potential in the prepubertal males could be assessed by measurements of testicular size. Normal pubertal development of males is expected because the absence of germ cells does not interfere with puberty, and in postpubertal males, follicle stimulating hormone and inhibin B levels, and sperm counts could be measured. In prepubertal girls anti-Mullerian hormone levels [22] should be measured to assess the ovarian reserve and data should be obtained on puberty, menarche, the regularity of subsequent menses, and subsequent fertility to evaluate the overall reproductive potential. The present study's results underscore the urgency of testing these reproductive parameters in boys and girls so that patients who will likely be given CP can be properly counseled about the potential risks of the drug to their unborn children, and appropriate measures taken to reduce any consequences.

## Supporting Information

Figure S1 Four-week-old 129 mice exposed to cyclophosphamide (7.5 mg/kg) on embryonic days 10.5 and 11.5 have kinks in their tails (arrows), a known teratogenic effect.
(PDF)

Table S1 Comparison of testicular germ cell tumor (TGCT) incidence and size in two sublines of 129 mice exposed to radiation or cyclophosphamide (CP) *in utero* on embryonic days 10.5 and 11.5.
(DOCX)

Table S2 Comparison of increase in the calculated number of tumor foci per testis in 129 and L1 mice exposed to cyclophosphamide (CP) or radiation *in utero*.
(DOCX)
