# Supplementary material for: Fetal Cyclophosphamide Exposure Induces Testicular Cancer and Reduced Spermatogenesis and Ovarian Follicle Numbers in Mice
Source: PLoS One. 2014 Apr 1;9(4):e93311. doi: 10.1371/journal.pone.0093311 (PMC3972108; doi:10.1371/journal.pone.0093311)
Supplement: Figure S1 — Four-week-old 129 mice exposed to cyclophosphamide (7.5 mg/kg) on embryonic days 10.5 and 11.5 have kinks in their tails (arrows), a known teratogenic effect. (PDF) [file pone.0093311.s001.pdf]

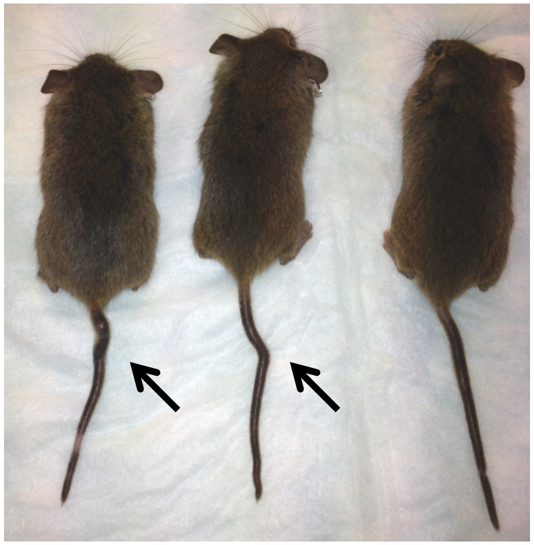

Figure. S1. Four-week-old 129 mice exposed to cyclophosphamide (7.5 mg/kg) on embryonic days 10.5 and 11.5 have kinks in their tails (arrows), a known teratogenic effect.
