# Supplementary material for: Fetal Cyclophosphamide Exposure Induces Testicular Cancer and Reduced Spermatogenesis and Ovarian Follicle Numbers in Mice
Source: PLoS One. 2014 Apr 1;9(4):e93311. doi: 10.1371/journal.pone.0093311 (PMC3972108; doi:10.1371/journal.pone.0093311)
Supplement: Table S1 — Comparison of testicular germ cell tumor (TGCT) incidence and size in two sublines of 129 mice exposed to radiation or cyclophosphamide (CP) in utero on embryonic days 10.5 and 11.5. (DOCX) [file pone.0093311.s002.docx]

Table S1.Comparison of testicular germ cell tumor (TGCT) incidence and size in two sublines of 129 mice exposed to radiation or cyclophosphamide (CP) *in utero* on embryonic days 10.5 and 11.5.

| Mouse  strain | Treatment | Body weight (g) | Incidence of tumors per mouse | | |  | Incidence of tumors per testis | | |  | Testis weight (mg) | |
| --- | --- | --- | --- | --- | --- | --- | --- | --- | --- | --- | --- | --- |
|  |  |  | No. males analyzed | Overall TGCT ^a^ | Bilateral TGCT ^a^ |  | No. testes analyzed | TGCT ^a^ | Identified as teratomas ^a^ |  | Testis with no TGCT | Testis with TGCT |
| 129S5 | Control ^b^ | 15.8 ± 0.6 | 30 | 1 (3%) | 0 (0%) |  | 60 | 1 (2%) | 1 (100%) |  | 61 ± 1 | 85 ± 0 |
|  | CP | 13.7 ± 0.6 ^c^ | 21 | 9 (43%) ^c^ | 3 (14%) |  | 42 | 12 (29%) ^c, d^ | 9 (75%) |  | 43± 2 ^c^ | 82 ± 16 |
|  | Radiation | 14.5 ± 0.4 | 22 | 6 (27%) ^c^ | 0 (0%) |  | 44 | 6 (14%) ^c^ | 6 (100%) |  | 38 ± 1^c^ | 93 ± 26 |
| 129S1  /SvImJ | Control ^b^ | 15.4± 0.2 | 52 | 1(2%) | 0 (0%) |  | 104 | 1 (1%) | 1(100%) |  | 56 ± 1 | 65 |
|  | CP | 12.9 ± 0.5 ^c^ | 32 | 6 (19%) ^c^ | 1 (3%) |  | 64 | 7 (11%) ^c, d^ | 4 (57%) |  | 41 ± 1^c^ | 99 ± 18 |
|  | Radiation | 14.1 ± 0.4 ^c^ | 12 | 5(42%) ^c^ | 0 (0%) |  | 24 | 5 (21%) ^c^ | 3 (60%) |  | 38 ± 3^c^ | 67 ± 15 |

^a^ Values given as absolute number and percentage of mice, testes, or tumors analyzed.

_b_ For each mouse substrain, the data of the radiation-control and CP-control mice were pooled because these two control groups’data were not significantly different except that the body weights of the 129S5 radiation-control mice were significantly heavier (17.7±0.6) than those of CP-control mice (13.4 ± 0.6 g; *P*<0.05).

^e^ Significantly different between treated and control mice (*P*<0.05; Fisher’s exact test or *t*-test as appropriate).

^d^ Significantly different between the two sublines of CP- treated mice (*P*=0.04; Fisher’s exact test).
