# Supplementary material for: Fetal Cyclophosphamide Exposure Induces Testicular Cancer and Reduced Spermatogenesis and Ovarian Follicle Numbers in Mice
Source: PLoS One. 2014 Apr 1;9(4):e93311. doi: 10.1371/journal.pone.0093311 (PMC3972108; doi:10.1371/journal.pone.0093311)
Supplement: Table S2 — Comparison of increase in the calculated number of tumor foci per testis in 129 and L1 mice exposed to cyclophosphamide (CP) or radiation in utero. (DOCX) [file pone.0093311.s003.docx]

| Mouse  strain | Treatment | Number of testes analyzed | Number of testes with TGCT | Probablitiy of TGCT in testis [P(t)] | Number of foci/ testis (μ) ^a^ | Number of foci, increase from control | No. foci L1/129 |
| --- | --- | --- | --- | --- | --- | --- | --- |
| 129 | Control | 164 | 2 ^b^ | 0.012 ^b^ | 0.012 ^b^ |  |  |
|  | CP | 106 | 19 | 0.179 | 0.198 | 0.185 |  |
|  | Radiation | 68 | 11 | 0.162 | 0.176 | 0.164 |  |
| L1 | Control | 92 | 18 | 0.196 | 0.218 |  | 17.7 ^b^ |
|  | CP | 39 ^c^ | 25 | 0.641 | 1.025 | 0.807 | 5.2 |
|  | Radiation | 64 | 51 | 0.797 | 1.594 | 1.376 | 9.0 |

Table S2.Comparison of increase in the calculated number of tumor foci per testis in 129 and L1 mice exposed to cyclophosphamide (CP) or radiation *in utero*

Abbreviation: TGCT, testicular germ cell tumor

^a^ The mean number of tumor foci/testis (μ) was calculated from the probability that there will be a tumor in the testis [P(t)]. The numbers of foci in the testes (k) will follow Poisson distribution with P(k) = μ^k^×e^-μ^/k!. Therefore, the probability of testis not having a tumor in the testis is P(0) = e^-μ^ , and the probability of a testis having tumor is P(t) = 1-P(0) = 1- e^-μ^. Therefore, the mean number of tumor foci/testis is μ = -ln[1-P(t)].

^b^ The number of TGCT bearing testes in this control group was lower than expected based on historical data from studies of 129 mice indicating a teratoma incidence of 5% in these mice (i.e. an incidence of 2.5% in these mice’s testes). Using this historical data, the mean number of tumor foci would be 0.025 and the ratio of number of foci per testis of L1 mice to that of 129 mice would be 8.6.

^c^ The odd number is due to the presence of only one testis in one of the mice analyzed.
